# Cutaneous Malignancies Metastatic to the Female Genital Tract and Pelvic Lymph Nodes: Analysis of Metastatic Patterns and Pathogenesis

**DOI:** 10.3390/jcm15145541

**Published:** 2026-07-15

**Authors:** Guglielmo Stabile, Laura Vona, Erika Pelaccia, Stefania Carlucci, Anna Pitsillidi, Mark Formosa, Marco Paratore, Luigi Nappi

**Affiliations:** 1 Department of Medical and Surgical Sciences, Institute of Obstetrics and Gynaecology, University of Foggia, 71122 Foggia, Italy; guglielmost@gmail.com (G.S.); s.carlucci86@gmail.com (S.C.); luigi.nappi@unifg.it (L.N.); 2 Department of Obstetrics and Gynecology, University of Chieti, 66100 Chieti, Italy; pelacciaerika@gmail.com; 3 Department of Obstetrics and Gynaecology, Rheinland Klinikum Neuss, Preußenstrasse 84, 41464 Neuss, Germany; anna.pitsillidi@gmail.com; 4 Department of Gynaecology and Obstetrics, Mater Dei Hospital, 2090 Msida, Malta; markf@maltanet.net; 5 Department of Obstetrics and Gynecology, IRCCS Ospedale Policlinico San Martino, 16132 Genoa, Italy; marcoparatore.94@gmail.com

**Keywords:** cutaneous melanoma, cutaneous squamous cell carcinoma, basal cell carcinoma, Merkel cell carcinoma, cutaneous adnexal carcinoma, cutaneous lymphoma, female genital tract

## Abstract

**Background/Objectives**: Metastases from cutaneous malignancies to the female genital tract and pelvic lymph nodes are rare clinical entities that frequently masquerade as primary gynecologic tumors, leading to significant diagnostic challenges. The distinction between primary and metastatic disease is critical, yet complex, given the varying patterns of spread exhibited by different skin cancers. This study aims to provide a tumor-specific overview of these metastatic patterns to guide diagnosis and therapy. **Methods**: We conducted a narrative review informed by a systematic literature search of MEDLINE/PubMed, Embase, Scopus, and Web of Science for records regarding primary cutaneous melanoma, cutaneous squamous cell carcinoma (cSCC), basal cell carcinoma (BCC), Merkel cell carcinoma (MCC), and cutaneous lymphomas metastasizing to the female genital tract (FGT) or pelvic lymph nodes. Data were synthesized qualitatively to identify organotropic patterns, diagnostic pitfalls, and management outcomes across these distinct malignancies. **Results**: The analysis reveals distinct metastatic niches: cutaneous melanoma shows a predilection for the ovary, often mimicking epithelial ovarian carcinoma, whereas cSCC and MCC typically involve pelvic lymph nodes via contiguous spread from inguinal basins. Histologic evaluation with broad immunohistochemical panels is mandatory to confirm the diagnosis, as imaging alone lacks specificity. Crucially, the introduction of immune checkpoint inhibitors and targeted therapies has significantly improved survival in advanced melanoma, cSCC, and MCC, altering the role of pelvic surgery. **Conclusions**: Management of cutaneous malignancies metastatic to the pelvis is shifting from a focus on radical surgery to a systemic-first approach. Pelvic metastasectomy should be reserved for selected oligometastatic cases or symptom control within a multidisciplinary framework. Clinicians must maintain a high index of suspicion in patients with a history of skin cancer to avoid overtreatment and optimize quality of life.

## 1. Introduction

Cutaneous malignancies, particularly melanoma, cutaneous squamous cell carcinoma (cSCC), and basal cell carcinoma (BCC), account for a substantial proportion of cancer diagnoses worldwide [1]. The IARC provides a worldwide estimate of approximately 1.2 million incident NMSC cases for the year 2022. In the United Kingdom and Ireland, data from the National Cancer Intelligence Network indicate that NMSCs represent 20% of all newly diagnosed cancers and 90% of skin malignancies. Within this group, basal cell carcinoma (BCC) is predominant, comprising around 74–75% of NMSCs and therefore representing the most frequent cancer by incidence [1,2,3,4,5,6,7,8]. Melanoma, despite its lower incidence compared with non-melanoma skin cancers, is responsible for most of the skin-cancer mortality due to its high metastatic potential and capacity for late, unpredictable visceral relapse [1,2]. cSCC and BCC are far more common but usually follow a more indolent course, with metastases occurring in a minority of high-risk cSCC and in an even smaller fraction of BCC cases [4,5,6,7,8].

The typical metastatic pattern of cutaneous malignancies involves regional lymph nodes and distant organs such as lung, liver, brain, bone, and skin [1,2,3]. In contrast, metastases to the female genital tract including ovaries, fallopian tubes, uterine corpus, cervix, vagina, vulva, and adnexa—and to pelvic lymph nodes are rare and often unexpected. Secondary ovarian malignancies account for approximately 5–7% of all ovarian cancers, most commonly arising from gastrointestinal and breast primaries; melanoma and other skin cancers constitute a small but distinct subset of these lesions [9,10,11,12].

Clinically, pelvic metastases from skin cancers frequently mimic primary gynecologic tumors. Ovarian metastases may present as solid adnexal masses indistinguishable from epithelial ovarian carcinoma on imaging; uterine metastases can be misdiagnosed as leiomyomas or primary endometrial tumors; cervical and vaginal lesions may be interpreted as primary mucosal melanoma or squamous carcinoma [9,10,11,12,13,14,15,16,17,18,19,20]. These diagnostic uncertainties have become even more relevant in the era of highly effective systemic therapies—immune checkpoint inhibitors, targeted agents, and, for selected tumors, hedgehog pathway inhibitors—which have significantly improved survival in advanced melanoma, cSCC, BCC, and Merkel cell carcinoma [4,5,6,7,8,21,22,23,24,25,26].

Previous reports have mostly consisted of isolated case reports and small series, often focusing on a single organ or single primary tumor type [4,5,6,9,10,11,12,13,14,15,16,17,18,19,20,25,26,27,28,29,30,31]. To our knowledge, no prior review has aggregated the literature across all major cutaneous malignancies with female genital tract and pelvic nodal metastases.

This review aims to provide a tumor-specific overview of metastatic patterns from cutaneous primary cancers to the female genital tract and pelvic lymph nodes, examining potential mechanisms of organotropism and tumor–microenvironment interactions in order to define diagnostic and therapeutic approaches. Given the rarity of these metastatic presentations and the heterogeneous nature of the available evidence, a narrative synthesis rather than a quantitative systematic review was considered the most appropriate methodological approach.

## 2. Materials and Methods

### 2.1. Search Strategy

This study is a narrative review supported by a systematic literature search conducted according to selected PRISMA 2020 recommendations for reporting the identification and selection process (Table S1—PRISMA Checklist). The available evidence regarding primary cutaneous malignancies metastatic to the female genital tract and pelvic lymph nodes was identified, screened, and synthesized qualitatively. Given the rarity of these metastatic presentations and the predominance of case reports and small retrospective series, a formal meta-analysis was not considered appropriate.

**Figure 1:**
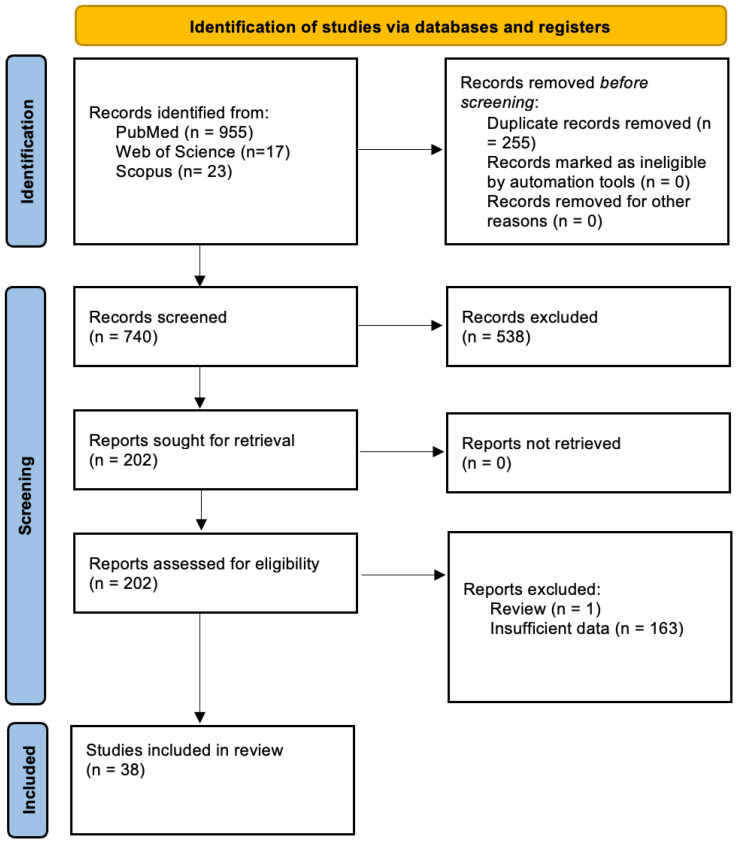
PRISMA Flow chart of study selection.

The identification, screening, eligibility assessment, and inclusion of studies were reported according to the PRISMA 2020 flow diagram recommendations (Figure 1). We searched MEDLINE/PubMed, Embase, Scopus, and Web of Science from database inception to March 2026 using combinations of MeSH and free-text terms related to cutaneous malignancies and pelvic/female genital tract (FGT) metastases: (cutaneous melanoma OR squamous cell carcinoma OR basal cell carcinoma OR Merkel cell carcinoma) AND (ovarian metastasis OR uterine metastasis OR vaginal metastasis OR vulvar metastasis OR pelvic lymph node) [1,2,3,4,5,6,7,8,9,10,11,12,25,26,27,28,29,30,31,32,33]. Reference lists of relevant articles and reviews were screened for additional reports.

Titles and abstracts were screened independently by at least two reviewers to identify potentially eligible articles. The full texts of selected records were then assessed in detail against the inclusion and exclusion criteria. Discrepancies at either stage were resolved by consensus or by consultation with a third reviewer.

This review was not prospectively registered in PROSPERO. Given the narrative nature of the review, the absence of a planned quantitative synthesis, and the predominance of case reports and small retrospective series in the available literature, prospective protocol registration was not considered applicable.

### 2.2. Eligibility Criteria

Inclusion criteria were as follows: primary cutaneous melanoma, cutaneous squamous cell carcinoma, basal cell carcinoma, cutaneous adnexal carcinomas (e.g., eccrine/apocrine carcinomas, porocarcinoma), Merkel cell carcinoma, or primary cutaneous lymphomas with secondary pelvic involvement; metastatic sites within the female genital tract (ovary, fallopian tube, uterine corpus, leiomyomas, cervix, vagina, vulva, adnexa) and/or pelvic lymph nodes (external, internal, and common iliac, obturator, presacral nodes). Eligible study designs included case reports, randomized controlled trials, prospective controlled studies, prospective cohort studies, retrospective studies, case series and systematic reviews containing primary clinical data [4,5,6,9,10,11,12,13,14,15,16,17,18,19,20,25,26,27,28,29,30,31]. Only full-text articles published in English were included. Articles not published in English, systematic reviews without primary clinical data, meta-analysis, letters to the editor, and conference abstracts were excluded.

However, reference lists of relevant reviews were manually screened to identify additional eligible studies. Exclusion criteria were primary mucosal melanomas or primary gynecologic carcinomas, isolated inguinal or retroperitoneal nodal metastases without pelvic nodal involvement, non-human studies, and reports lacking sufficient detail to confirm cutaneous origin and pelvic site of metastasis.

### 2.3. Data Extraction and Synthesis

Given the marked heterogeneity of the available literature and the predominance of isolated case reports and small retrospective series, formal risk-of-bias assessment tools were not systematically applied. Instead, reporting quality was qualitatively evaluated using selected elements of the CARE guidelines. For larger series and cohort studies, we noted key design features and limitations [4,5,6,9,10,11,12,27,28,29,30,31]. Duplicate records were manually identified and removed independently by two reviewers (E.P. and L.V.). Data were synthesized descriptively and organized primarily by primary neoplasia: melanoma (Section 3), cSCC (Section 4), BCC (Section 5), Merkel cell and adnexal carcinomas (Section 6), and cutaneous lymphomas (Section 7). For each tumor type, we summarize patterns of spread, pathogenesis, diagnostic work-up, and management.

## 3. Cutaneous Melanoma

**Figure 2:**
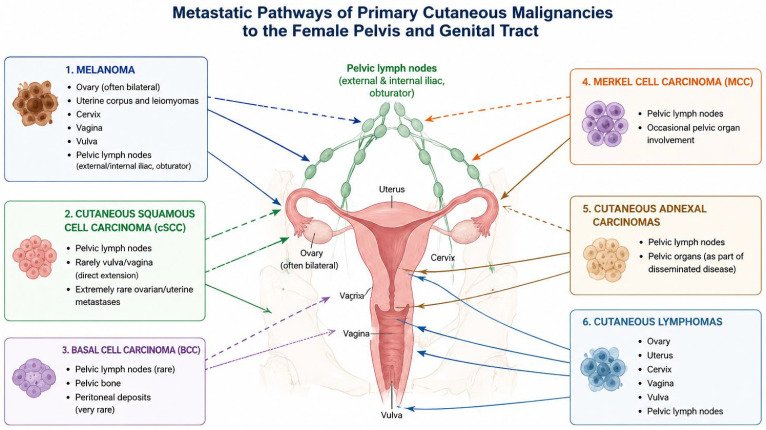
Metastatic patterns from primary cutaneous malignancies to the female genital tract and pelvic lymph nodes.

Melanoma is a highly aggressive malignancy with a strong propensity for both lymphatic and hematogenous metastasis [1,2,34]. Autopsy series from the pre-immunotherapy era showed frequent abdominal and pelvic involvement in women dying of disseminated melanoma, with ovarian metastases observed in up to 15–20% of cases in some large series [1,2,3,9,10,11,12]. Clinically recognized metastases to the female genital tract are much less frequent and tend to be reported as isolated cases or small series [9,10,11,12,13,14]. Abdominal–pelvic CT series have demonstrated that in patients with metastatic melanoma, the abdomen and pelvis are commonly involved, with the liver and pelvic lymph nodes among the most frequent sites of metastasis [3]. In such cohorts, pelvic nodal disease often coexists with visceral metastases, including ovarian and uterine involvement [3]. An overview of the patterns of metastatic spread from each primary cutaneous malignancy to the female genital tract and pelvic lymph nodes is provided in Table 1 and Figure 2.

### 3.1. Female Genital Tract and Pelvic Lymph Node Involvement

The ovary is the most frequent genital site for metastatic melanoma. In the largest clinicopathologic series, virtually all ovarian melanomas represent metastases rather than primary tumors, except for rare melanomas arising within mature cystic teratomas [10,11]. Ovarian metastases may derive from cutaneous, ocular, or mucosal primaries, but cutaneous melanoma predominates [9,10,11,12,13]. Clinically, ovarian metastases occur over a wide age range, including premenopausal women, are often bilateral, and present as solid or solid-cystic adnexal masses, frequently with ascites and elevated CA-125, mimicking epithelial ovarian carcinoma [9,10,11,12,13,14].

Uterine metastases are less common and may involve the myometrium, serosa, or endometrium, sometimes colonizing pre-existing leiomyomas as tumor-to-tumor metastases [15,16,17]. Cervical and vaginal lesions are exceptionally rare but have been reported, often presenting with abnormal bleeding or being detected on routine cytology [18,19,20]. Vulvar metastases can be difficult to distinguish from primary mucosal melanomas [19,20]. Pelvic lymph node involvement is frequently observed in patients with lower extremity or perineal primaries, typically as extension of inguinal nodal disease [3].

### 3.2. Management and Outcomes

Systemic therapy is the backbone of treatment for metastatic melanoma. Immune checkpoint inhibitors (anti–PD-1 alone or combined with anti–CTLA-4) have significantly improved survival in advanced melanoma, with durable responses in a substantial proportion of patients [21,22,23,24]. Targeted therapies with BRAF and MEK inhibitors are highly effective in BRAF V600–mutant melanoma, often yielding rapid tumor shrinkage [24].

Pelvic surgery is considered in selected scenarios, such as symptomatic pelvic disease (pain, bleeding, obstruction), isolated or oligometastatic disease amenable to complete resection, or residual localized pelvic disease in patients responding well to systemic therapy [1,2,3,34,35]. Evidence from retrospective metastasectomy series suggests that complete resection of isolated abdominal metastases, including ovarian and uterine lesions, may be associated with improved outcomes in carefully selected patients, particularly when integrated with modern systemic therapy [1,2,3,34,35].

Prognosis depends primarily on overall metastatic burden, performance status, and response to systemic therapy rather than on genital involvement per se [1,2,3,21,22,23,24].

## 4. Cutaneous Squamous Cell Carcinoma (cSCC)

Cutaneous Squamous Cell Carcinoma (cSCC) is the second most common skin cancer after BCC. Most cases are cured with local therapy, but a subset exhibits aggressive behavior with regional and distant metastases [4,5,6,7,8]. High-risk features include large size, deep invasion, poor differentiation, perineural or lymphovascular invasion, and immunosuppression [7,8]. Pelvic involvement most often arises via lymphatic spread from cutaneous primaries of the lower extremities, buttocks or perineum [7,8,32,33].

### 4.1. Pelvic Lymph Node and Genital Tract Involvement

Pelvic nodal metastases from cSCC are typically a continuation of inguinal nodal disease. Data from vulvar carcinoma provide a useful model: pelvic node involvement is strongly associated with multiple positive inguinal nodes, large nodal metastases, and extracapsular extension, and is a major adverse prognostic factor [32,33]. Although these data refer to vulvar squamous carcinoma, analogous drainage patterns apply to perineal and lower extremity cSCC [7,8,32,33].

Direct invasion of vulva, vagina, or lower rectum from locally advanced perineal cSCC is more common than true hematogenous metastasis to ovaries or uterus. Reports of isolated cSCC metastases to intrapelvic gynecologic organs without contiguous spread are exceedingly rare [32,33].

### 4.2. Management

Management of advanced cSCC relied on surgery and radiotherapy. The advent of anti–PD-1 immunotherapy has significantly changed the paradigm. Cemiplimab and other anti–PD-1 antibodies are now standard-of-care for unresectable locally advanced or metastatic cSCC, with response rates of approximately 40–50% and durable disease control in many patients [7,8,28,29]. Neoadjuvant immunotherapy is increasingly used to downstage bulky nodal disease, including pelvic involvement, potentially permitting less extensive surgery [7,8,29]. For pelvic nodal cSCC, the limited available evidence and contemporary treatment paradigms support consideration of a strategy that prioritizes systemic immunotherapy, followed by restaging and individualized evaluation of surgery or radiotherapy for residual pelvic disease. Radical pelvic lymphadenectomy or exenteration is rarely justified unless disease is strictly localized and systemic staging is otherwise negative [7,8,32,33].

## 5. Basal Cell Carcinoma

BCC is the most common human malignancy but has an extremely low metastatic potential [4,5,6]. Systematic reviews estimate metastasis rates between 0.0028% and 0.55%, typically in association with large, long-standing, or multiply recurrent tumors, often of the head and neck [4,5,6]. Most metastatic BCCs involve regional nodes, lung, bone, and distant skin or soft tissue; pelvic and FGT metastases are exceptional [4,5,6].

### 5.1. Pelvic and Genital Tract Metastases

Reports of BCC involving the pelvis or FGT describe iliac or obturator nodal metastases, usually in continuity with inguinal nodes, in the setting of advanced regional disease; and rare peritoneal or visceral pelvic deposits in widely disseminated disease [4,5,6]. True metastases to the ovary, uterus, cervix, vagina, or vulva from BCC are extraordinarily rare; most vulvar BCCs are primary lesions, typically indolent and confined to the vulvar skin [6].

### 5.2. Diagnostic and Therapeutic Considerations

Given the rarity of pelvic BCC metastases, diagnosis requires histologic confirmation with classic BCC morphology and immunohistochemistry (e.g., Ber-EP4 positivity) to distinguish BCC from other basaloid tumors [6]. Systemic treatment of metastatic BCC centers on hedgehog pathway inhibitors such as vismodegib and sonidegib, which can achieve meaningful responses in many patients [4,5,6]. For those who progress or are intolerant, emerging data support the use of immunotherapy in select cases [4,5]. Pelvic surgery is generally limited to palliative interventions for symptomatic nodal or osseous disease.

## 6. Merkel Cell Carcinoma and Cutaneous Adnexal Carcinomas

MCC is a rare, aggressive neuroendocrine carcinoma of the skin, frequently associated with Merkel cell polyomavirus and/or chronic UV exposure [25,26]. It carries high rates of nodal and distant metastases and poor prognosis [25,26]. Lower extremity or buttock MCCs often drain to inguinal and then pelvic nodes, mirroring melanoma and cSCC [25,26]. Pelvic nodal disease appears largely as an extension of inguinal involvement, and FGT structures can be secondarily involved by direct extension or as part of disseminated disease.

### 6.1. Merkel Cell Carcinoma

Pathologically, MCC is characterized by small, round blue cells with neuroendocrine granules and a distinctive immunophenotype (CK20-positive in a perinuclear dot pattern, synaptophysin-positive, chromogranin-positive, TTF-1–negative), helping distinguish it from small-cell lung carcinoma [25,26].

Management of metastatic MCC has been transformed by immunotherapy. Avelumab, pembrolizumab, and nivolumab are established agents for advanced MCC with high response rates and durable remissions in a subset of patients [25,26]. Surgery and radiotherapy play roles in local control but should be integrated with systemic therapy rather than used in isolation. Based on the limited available literature and current systemic treatment approaches, pelvic surgery for MCC is often restricted to diagnostic biopsies or symptom-relieving procedures [25,26].

### 6.2. Cutaneous Adnexal Carcinomas

Cutaneous adnexal carcinomas (e.g., eccrine porocarcinoma, hidradenocarcinoma, primary cutaneous adenoid cystic carcinoma) are rare but often aggressive tumors with significant metastatic potential. Pelvic involvement typically occurs via nodal spread from lower extremity or perineal primaries or as part of systemic dissemination [7,8]. Diagnostic work-up relies heavily on pathology and immunohistochemistry to classify the adnexal tumor and distinguish it from metastatic visceral carcinomas. Management is individualized, often involving wide local excision, regional lymph node dissection, and systemic therapy tailored to histologic subtype [7,8].

## 7. Cutaneous Lymphomas

Primary cutaneous lymphomas, including mycosis fungoides, Sézary syndrome, and various B-cell lymphomas, may secondarily involve lymph nodes and extranodal organs [27,28,29,30,31]. The female genital tract is an uncommon but recognized site of lymphoma, with cases involving ovary, uterus, cervix, vagina, and vulva described in institutional series and reviews [27,28,29,30,31]. Some of these represent primary gynecologic lymphomas; others reflect systemic or cutaneous lymphomas with secondary reproductive tract involvement [27,28,29,30,31].

### 7.1. Patterns of Spread and FGT Involvement

In women with known cutaneous lymphoma, FGT lesions may present as vaginal or vulvar masses, ulcers, or plaques, as uterine or ovarian masses on imaging, or as pelvic lymphadenopathy [27,28,29,30,31]. Distinguishing primary gynecologic lymphoma from secondary involvement often requires systemic staging, including PET/CT and bone marrow biopsy, and careful correlation with prior cutaneous or nodal disease [28,29,30,31].

### 7.2. Treatment

Management of FGT involvement by cutaneous or systemic lymphoma is systemic and subtype-driven. Diffuse large B-cell lymphoma-type lymphomas are typically treated with R-CHOP or related regimens; indolent B-cell lymphomas are treated with immunochemotherapy or targeted agents as indicated; and T-cell lymphomas require individualized regimens, often including combination chemotherapy and skin-directed therapies [28,29,30,31]. Radical gynecologic surgery is rarely reported in this setting and is unlikely to provide benefit in most cases, although management should remain individualized according to lymphoma subtype and disease extent; limited excision may be used for diagnosis or symptom control, but definitive management is hematologic [28,29,30,31].

## 8. General Diagnostic Principles and Multidisciplinary Management

Although previous sections address tumor-specific features, several cross-cutting principles apply to all cutaneous malignancies metastatic to the FGT and pelvic nodes. In any woman with a current or past history of melanoma or high-risk non-melanoma skin cancers who presents with a pelvic mass, abnormal bleeding, or pelvic lymphadenopathy, metastatic disease should be considered [1,2,3,4,5,6,7,8,25,26,32,33,34,35,36]. A detailed oncologic history, full skin examination, and careful review of prior pathology and imaging are essential. Advanced imaging with pelvic MRI, CT, and PET/CT is crucial to assess the extent of pelvic and systemic disease, recognizing physiological pelvic FDG uptake in premenopausal women [3,37,38]. Histologic confirmation with an appropriately broad immunohistochemical panel is mandatory before major surgery [6,7,8,9,10,15,16,17,18,19,20,25,26,27,28,29,30,31]. Whenever possible, management should be discussed in a multidisciplinary tumor board integrating dermatologic oncology, gynecologic oncology, medical oncology, radiation oncology, radiology, nuclear medicine, and pathology.

## 9. Pathology and Immunohistochemistry

Histologic evaluation is central to distinguishing metastatic cutaneous malignancies from primary gynecologic tumors. In melanoma, metastatic deposits in the ovary and uterus typically show epithelioid or spindle-cell morphology with variable melanin pigmentation and high mitotic activity, while cSCC presents as infiltrative nests of atypical squamous cells with keratinization, and MCC as sheets of small blue cells with neuroendocrine features [1,2,3,7,8,9,10,15,16,17,18,19,20,25,26].

Basal cell carcinoma displays basaloid nests with peripheral palisading and retraction artifact, whereas lymphomas efface native architecture with diffuse or nodular lymphoid infiltrates [4,5,6,27,28,29,30,31]. A broad immunohistochemical panel tailored to the suspected lineage is essential, and typical profiles for the main differential diagnoses are summarized in Table 2. In particular, melanocytic markers (S100, SOX10, Melan-A, HMB-45), epithelial markers (broad cytokeratins, PAX8, WT1), squamous markers (p40, p63), neuroendocrine markers (CK20, synaptophysin, chromogranin), and lymphoid markers (CD3, CD20, light-chain restriction) should be applied as appropriate, together with HPV-related biomarkers in anogenital squamous lesions [6,7,8,9,10,15,16,17,18,19,20,25,26,27,28,29,30,31].

Molecular testing may provide additional diagnostic support in selected cases. In melanoma, concordance of driver mutations such as BRAF V600 between the primary cutaneous lesion and the pelvic tumor may support a metastatic relationship and may also guide targeted therapy decisions [24]. In squamous lesions, HPV status assessment may help distinguish primary HPV-related cervical or anogenital carcinomas from metastatic cutaneous squamous cell carcinoma, which is generally HPV-negative [6,7,8,9,10]. Similarly, assessment of Merkel cell polyomavirus status may support the diagnosis of metastatic cutaneous Merkel cell carcinoma and help differentiate it from other primary neuroendocrine malignancies of the gynecologic tract [25,26].

## 10. Discussion

This review synthesizes the highly fragmented literature on cutaneous malignancies metastatic to the female genital tract (FGT) and pelvic lymph nodes, organizing the available evidence according to the biology of the primary neoplasm [1,2,3,4,5,6,7,8,9,10,11,12,25,26,27,28,29,30,31,32,33].

Metastases from cutaneous malignancies to the female genital tract and pelvic lymph nodes represent a small but clinically significant corner of gynecologic oncology [1,2,3,4,5,6,7,8,9,10,11,12,25,26,27,28,29,30,31,32,33]. Their rarity belies their importance, because they tend to occur in patients with complex oncologic histories and because they sit at the intersection of dermatology, medical oncology, radiology, pathology, and gynecologic surgery. This review shows that these metastases cannot be understood as a single entity: each primary skin cancer brings its own pattern of organotropism, route of spread, diagnostic pitfalls, and therapeutic vulnerabilities [1,2,3,4,5,6,7,8,25,26,27,28,29,30,31,32,33,34,35].

At a molecular level, these differences in metastatic behavior likely reflect interactions between tumor-intrinsic properties and organ-specific microenvironments. The ovary, particularly in melanoma, may represent a permissive hematogenous “soil” characterized by rich vascularization, stromal support, and chemokine gradients that facilitate tumor cell adhesion and growth [39]. By contrast, pelvic lymph node involvement more commonly reflects stepwise lymphatic dissemination, in which tumor cells exploit established lymphatic drainage pathways and interact with immune cell-rich microenvironments that function both as filters and as potential metastatic niches [40,41]. These distinct biological contexts support the concept that the female genital tract and pelvic lymph nodes constitute biologically heterogeneous metastatic environments, where organ-specific vascular, stromal, and immune features likely contribute to differential patterns of metastatic colonization.

These metastatic patterns have important therapeutic implications. In melanoma, cSCC, and MCC, the rapid evolution of systemic therapies has fundamentally changed the timing and the way to operate in the pelvis [7,8,21,22,23,25,26,34,36,37]. Immune checkpoint inhibitors and targeted therapies have turned some stage IV melanomas into chronic or even potentially curable conditions, and anti–PD-1 therapy has similarly transformed the management of advanced cSCC and MCC [7,8,21,22,23,24,34,35,36,37]. In this new context, extensive pelvic surgery performed with curative intent through radical cytoreduction should be carefully weighed, as it may offer limited benefit when systemic disease remains uncontrolled [1,2,3,7,8,21,22,23,24,34,36,37]. The emerging paradigm, supported by case series and the broader literature on resection of metastases, suggests that surgery should be integrated with systemic therapy rather than considered in isolation [1,2,3,7,8,34,35,36,37,38].

For MCC and lymphomas, where systemic therapy represents the primary treatment modality, surgery is usually limited to biopsy and selected palliative procedures [25,26,27,28,29,30,31]. For women with a solitary ovarian melanoma metastasis after a long disease-free interval and otherwise controlled disease, removal of the adnexal lesion may confer both symptomatic relief and potential survival benefit, particularly if undertaken in conjunction with systemic therapy [9,10,11,12,13,21,22,23,24,34,35].

Finally, it is important to acknowledge the limitations of the evidence base synthesized. Most of the literature consists of single case reports or small retrospective series, selectively published because they are unusual or illustrative [4,5,6,9,10,11,12,13,14,15,16,17,18,19,20,27,28,29,30,31]. This introduces significant publication and selection bias. Critical variables such as genomic profiles, detailed prior treatment histories, and long-term outcomes are often missing. Heterogeneity in reporting makes it difficult to quantify incidence, identify robust prognostic factors, or compare different therapeutic strategies [4,5,6,9,10,11,12,27,28,29,30,31]. Nonetheless, even this imperfect evidence, when systematically assembled and interpreted through the lens of tumor biology and modern oncology, offers a coherent set of principles that can inform everyday clinical decision-making [1,2,3,7,8,21,22,23,24,25,26,27,28,29,30,31,34,35,36,37,38,42,43]. Furthermore, the available studies span different diagnostic and therapeutic eras, limiting the generalizability of reported outcomes to contemporary clinical practice. Consequently, many of the diagnostic and therapeutic considerations discussed in this review should be regarded as hypothesis-generating and interpreted with caution rather than as recommendations supported by high-level prospective evidence.

In women with a history of skin cancer who present with a newly diagnosed pelvic mass or pelvic lymphadenopathy, metastatic disease should be routinely considered in the differential diagnosis [1,2,3,4,5,6,7,8,9,10,11,12,25,32,33]. Appropriate evaluation includes careful documentation of dermatologic and oncologic history, retrieval of prior pathology and imaging, and communication of this information to radiologists and pathologists [3,6,7,8,9,10,15,16,17,18,19,20,25,26,27,28,29,30,31]. Given the potential for misleading morphologic and radiologic features, broad immunohistochemical profiling and, when feasible, molecular comparison with the primary cutaneous tumor may be required to establish the correct diagnosis [1,2,3,4,5,6,7,8,9,15,16,17,18,19,20,25,31,32,33,34,35,43].

In some cases, pelvic surgery will remain pivotal, particularly for isolated or oligometastatic disease or for control of severe symptoms [7,8,9,10,11,12,15,16,17,34,35,36,37,44,45]. In many others, however, optimal care will involve prioritizing systemic treatment, reserving surgery for focused, low-morbidity interventions, and relying on radiotherapy for local control when necessary [7,8,25,26,27,28,29,30,31,36,37].

**Figure 3:**
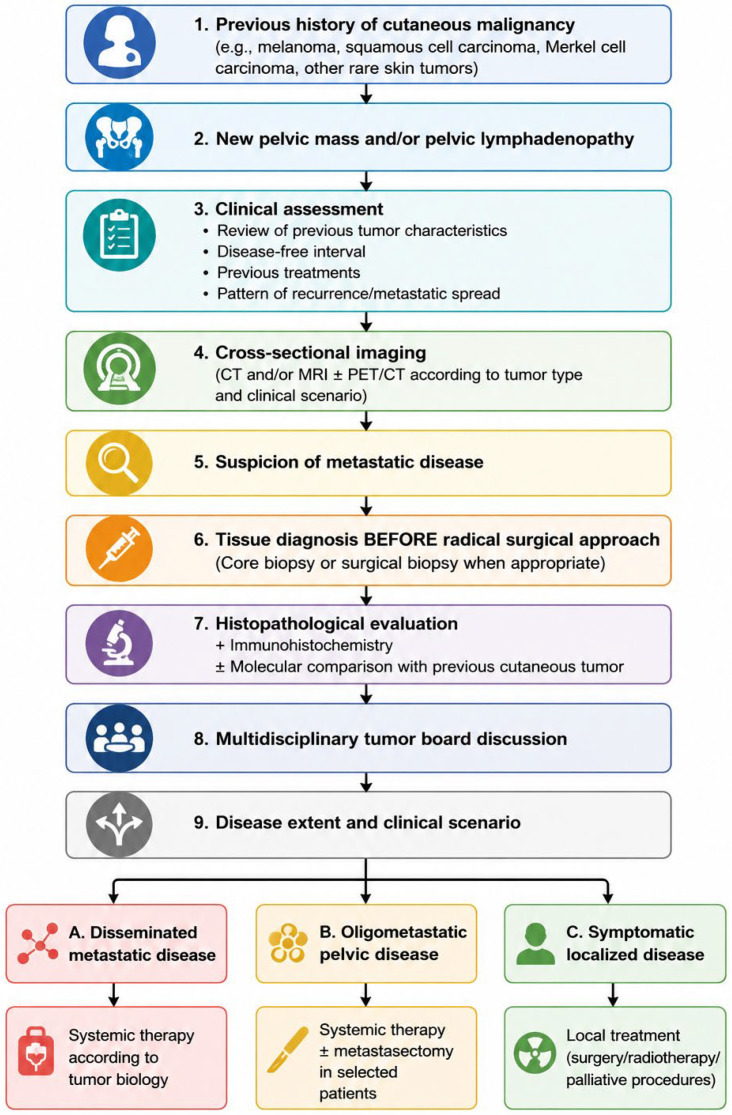
Proposed diagnostic and therapeutic algorithm for women with a history of cutaneous malignancy presenting with a pelvic mass or lymphadenopathy.

Based on the available evidence, we propose a pragmatic diagnostic and therapeutic algorithm to support the clinical management of women with a history of cutaneous malignancy presenting with pelvic lesions (Figure 3).

Looking ahead, progress will depend on collective rather than individual efforts. The creation of registries that systematically capture clinical, imaging, histopathologic, and molecular data on cutaneous malignancies metastatic to the FGT and pelvic nodes would greatly enhance our understanding of their true incidence, natural history, and response to modern therapies [1,2,3,4,5,6,7,8,9,10,11,12,25,26,27,28,29,30,31,32,33]. The integration of translational research into such registries could help clarify the molecular determinants of pelvic organotropism and identify biomarkers that predict which patients are at particular risk [1,2,34,35,43]. More generally, the inclusion of patients with pelvic and genital metastases in larger clinical trials and real-world studies of systemic therapies for melanoma, cSCC, BCC, MCC, and lymphomas would allow post hoc analyses of outcomes in this subgroup, providing much-needed evidence to guide the sequencing of systemic and local treatments [7,8,21,22,23,25,26,27,28,29,30,31,34,36,37].

Given the exceptional rarity of these metastatic presentations, prospective multicenter data collection using standardized reporting criteria should be encouraged. Harmonized datasets including clinical characteristics, imaging findings, histopathological features, molecular profiling, treatment strategies, and long-term outcomes would facilitate more robust comparisons across studies and provide the evidence needed to develop evidence-based diagnostic and therapeutic recommendations.

## 11. Limitations

Several limitations should be acknowledged when interpreting the findings of this review. The available literature on cutaneous malignancies metastatic to the female genital tract and pelvic lymph nodes is extremely limited and predominantly consists of case reports, small case series, and retrospective studies. This inevitably introduces a risk of publication bias and reporting bias, as rare and clinically challenging presentations are more likely to be published, while standardized clinical and pathological information is often unavailable. Moreover, the heterogeneity in diagnostic approaches, immunohistochemical assessment, staging procedures, treatments, and follow-up duration limits the comparability of reported cases and prevents reliable estimation of incidence or meaningful comparison of therapeutic strategies.

Although this review was based on a structured literature search and reported according to PRISMA recommendations, the rarity and heterogeneity of the available evidence did not allow quantitative synthesis or formal risk-of-bias assessment. Therefore, the conclusions should be interpreted as a synthesis of the current evidence to support clinical decision-making rather than as definitive treatment recommendations. Further multicenter registries and standardized collection of clinical, pathological, and molecular data are needed to improve the understanding and management of these rare metastatic presentations.

## 12. Conclusions

Pelvic and female genital tract metastases from cutaneous malignancies are rare but clinically consequential because they frequently mimic primary gynecologic tumors and may lead to overtreatment if misclassified [14,15,16,17,18,19]. Distinct metastatic routes emerge: melanoma preferentially involves the ovary and other genital organs, whereas cSCC and MCC more often reach pelvic lymph nodes via stepwise lymphatic spread from inguinal basins; BCC and cutaneous lymphomas represent exceptional scenarios that require entity-specific diagnostic and therapeutic approaches [4,5,6,7,8,9,10,28,29,30,31,32,33]. In the era of effective immunotherapy and targeted agents, management is increasingly shifting toward a systemic-first strategy, with pelvic surgery reserved for selected oligometastatic presentations, symptom control, or residual localized disease after systemic response [1,2,3,4,5,6,7,8,21,22,23,25,26,34,35,36,37,44].

Until more robust data become available, improving care for these patients requires maintaining awareness of metastatic skin cancer as a potential cause of pelvic disease, performing rigorous and integrated diagnostic work-ups, and basing management decisions on multidisciplinary, biology-driven discussions rather than organ-based assumptions [1,2,3,7,8,21,22,23,24,25,26,27,28,29,30,31,34,35,36,37,43,45,46,47]. This approach may reduce the risk of misdiagnosis and overtreatment, better align therapy with systemic disease biology, and ultimately improve both oncologic outcomes and quality of life for women affected by these rare metastases.

## Figures and Tables

**Table 1:** Summary of metastatic patterns from primary cutaneous malignancies to the female genital tract and pelvic lymph nodes.

Primary Cutaneous Malignancy	Typical Pelvic/FGT Metastatic Sites	Predominant Route of Spread	Usual Clinical Context
Melanoma	Ovary (often bilateral); uterine corpus and leiomyomas; cervix; vagina; vulva; pelvic lymph nodes (external/internal iliac, obturator).	Mainly hematogenous; lymphatic for nodal disease.	Long disease-free interval; prior cutaneous or ocular primary; often disseminated disease at detection.
Cutaneous squamous cell carcinoma (cSCC)	Pelvic lymph nodes; rarely vulva/vagina by direct extension; extremely rare ovarian/uterine metastases.	Lymphatic, stepwise from inguinal to pelvic basins.	High-risk primary on lower limb or perineum; heavy inguinal nodal burden; immunosuppression.
Basal cell carcinoma (BCC)	Pelvic lymph nodes (rare); pelvic bone; peritoneal deposits (very rare).	Lymphatic and hematogenous in exceptional cases.	Advanced, large, or neglected primary; often head and neck.
Merkel cell carcinoma (MCC)	Pelvic lymph nodes; occasional pelvic organ involvement.	Lymphatic spread from lower limb/buttock primaries, plus hematogenous.	Rapidly progressive nodal disease; frequent coexisting distant metastases.
Cutaneous adnexal carcinomas	Pelvic lymph nodes and organs as part of disseminated disease.	Mostly lymphatic, sometimes hematogenous.	Advanced, high-grade adnexal tumors; often lower extremity/perineal.
Cutaneous lymphomas	Ovary, uterus, cervix, vagina, vulva; pelvic lymph nodes.	Hematogenous and lymphatic.	Systemic or primary cutaneous lymphoma with secondary FGT involvement.

**Table 2:** Pathologic and immunohistochemical features distinguishing metastatic cutaneous tumors from primary gynecologic neoplasms.

Entity/Differential Diagnosis	Key Histologic Features	Immunohistochemical Profile (Typical)	Distinguishing Diagnostic Features
Metastatic cutaneous melanoma to ovary/uterus	Epithelioid/spindle cells; variable melanin; high mitotic activity; often no in situ component.	S100+, SOX10+, Melan-A+, HMB-45+; Cytokeratin−; WT1−; Inhibin−	History of melanoma; no junctional melanocytic proliferation in adjacent epithelium; may mimic epithelial or sex-cord tumors; concordant BRAF mutations between primary and metastatic lesions may support a metastatic relationship.
Primary ovarian epithelial carcinoma	Papillary/solid/glandular structures; high-grade atypia.	CK7+, PAX8+, WT1+ (In serous); Melan-A−, SOX10−	Often bilateral with surface involvement; no melanocytic markers.
Primary uterine/endometrial melanoma	Mucosal in situ component; junctional proliferation.	Similar to metastatic melanoma (melanocytic markers+); may be KIT-mutated	Extremely rare; requires absence of extra-uterine primary and demonstrable in situ change.
Metastatic cutaneous SCC to pelvic nodes	Nests of atypical squamous cells; keratinization; often extranodal spread.	P40+, p63+, Pan-cytokeratin+; p16 variable; HPV-negative	High-risk cutaneous primary; UV-signature mutations; typically HPV-negative; must be distinguished from HPV-driven anogenital SCC.
Primary cervical/vulvar SCC	Squamous dysplasia or in situ carcinoma; often HPV-associated.	P40+, p63+, p16+ (in HPV-related); HPV-DNA often detectable	Intraepithelial precursor often present; no history of high-risk cSCC.
Basal cell carcinoma metastatic	Basaloid nests; peripheral palisading; retraction artifact	Ber-EP4+, BCL-2+, EMA−/focal; cytokeratin+	Metastasis very rare; exclusion of other basaloid carcinomas is essential
Merkel cell carcinoma	Dermal/submucosal sheets of small blue cells	CK20+ (perinuclear dot), synaptophysin+, chromogranin+, TTF-1−	Differentiates from small-cell lung carcinoma (usually ck20−, ttf-1+); Merkel cell polyomavirus status may support cutaneous origin in selected cases.
Lymphomas involving FGT	Diffuse or nodular lymphoid infiltrates	Lineage-specific markers (CD20, CD3, etc.), light chain restriction, Ki-67 variable	Requires systemic staging; radical gynecologic surgery rarely indicated.

## Data Availability

The authors confirm that the data supporting the findings of this study are available within the article.

## Supplementary Materials

The following supporting information can be downloaded at: https://www.mdpi.com/article/10.3390/jcm15145541/s1, Table S1: PRISMA 2020 Checklist [48].

## Abbreviations

The following abbreviations are used in this manuscript:

cSCC	Cutaneous squamous cell carcinoma
BCC	Basal cell carcinoma
MCC	Merkel cell carcinoma
FGT	Female genital tract
NMSC	Non-melanoma skin cancers
